# A Photocatalytic TiO_2_ Coating with Optimized Mechanical Properties Shows Strong Antimicrobial Activity Against Foodborne Pathogens

**DOI:** 10.3390/ma18245640

**Published:** 2025-12-15

**Authors:** Eduardo Torres Domínguez, Fnu Chenggeer, Liang Mao, Matthew R. Maschmann, Heather K. Hunt, Azlin Mustapha

**Affiliations:** 1 Department of Chemical and Biomedical Engineering, University of Missouri, Columbia, MO 65211, USA; lalotd@gmail.com; 2 Food Science Program, University of Missouri, Columbia, MO 65211, USA; xcmq2@missouri.edu (F.C.); lmn7z@missouri.edu (L.M.); 3 Department of Mechanical and Aerospace Engineering, University of Missouri, Columbia, MO 65211, USA; maschmannm@missouri.edu

**Keywords:** antimicrobial coating, titanium dioxide, hardness, elastic modulus, wear, photocatalytic activity

## Abstract

Advanced technologies, such as antimicrobial coatings on food contact surfaces (FCSs), are critical to prevent the occurrence of food-contaminating bacteria. Titanium dioxide coatings were fabricated by the sol–gel method on stainless steel following an experiment consisting of eight different combinations of these synthetic parameters: type of protocol (method), amount of surfactant, aging time, spinning speed, and sintering temperature. Hardness and elastic modulus values of the eight coating combinations were assessed by nanoindentation, and their values were statistically analyzed to determine which protocol and sintering temperature were significant influencing factors. Additional experimental points were procured to obtain trends relating sintering temperature to hardness and elastic modulus. Within the experimental range studied, hardness monotonically increased with sintering temperature, reaching its maximum value at 595 °C, while elastic modulus attained a maximum value at 640 °C. These maxima’s isotherms were overlapped on the coating’s photocatalytic activity contour plot to explore which combinations of protocol, aging time, and sintering temperature yielded optimal photocatalytic activity, hardness, and elastic modulus. The optimized coating was tested against two representative foodborne pathogens, *Escherichia coli* O157:H7 and *Staphylococcus aureus* cells and their biofilms, and was characterized by nanoindentation, scanning electron microscopy, and X-ray diffraction. The properties of the coating, as found in this study, present evidence for its potential FCS applications.

## 1. Introduction

Foodborne pathogens, such as *Salmonella*, *Escherichia coli*, and *Listeria monocytogenes*, as well as antibiotic-resistant strains, cause a significant global health burden, resulting in approximately 600 million global foodborne illnesses and 420,000 deaths annually [1,2]. Contaminated food contact surfaces are a primary transmission vector, underscoring the need for effective and persistent sanitization methods beyond conventional cleaning, which can fail to prevent recontamination and biofilm formation [3,4,5,6,7,8,9].

Antimicrobial coatings, particularly photocatalytic titanium dioxide, offer a promising solution by providing continuous surface disinfection through the generation of reactive oxygen species (ROS), while avoiding issues of agent depletion and antibiotic resistance [6,10,11]. TiO_2_ coatings have demonstrated robust efficacy against various pathogens [12,13,14]. However, their practical application is critically limited by a lack of durability, with documented failures, including delamination and mechanical wear, that reduce functionality [6,15,16,17].

A fundamental conflict exists between the functional and structural properties of these coatings. Durability, defined as resistance to mechanical stress, operational loads, or degradation, depends heavily on deposition methods and processing parameters [18,19]. High photocatalytic activity, favored by a porous, anatase-rich structure, is often inversely related to mechanical robustness, which is enhanced by denser, rutile-rich phases and specific processing conditions [20,21,22,23,24,25,26]. This performance–durability trade-off represents a key barrier to deployment.

This work addresses this problem by systematically investigating the synthesis parameters for sol–gel TiO_2_ coatings to identify conditions that co-optimize mechanical and functional properties. The relationship between sintering temperature and the mechanical properties of hardness and elastic modulus was established. These trends were then integrated with a previously developed model for photocatalytic activity [26] to define a fabrication window that simultaneously enhances durability and antimicrobial performance. Coatings prepared under these optimized conditions were characterized and evaluated for efficacy against relevant foodborne pathogens.

The main aim of this study was to maximize durability, as measured by hardness and elastic modulus, while maximizing the photocatalytic activity of an antimicrobial coating. Effects of manufactured coatings on two important foodborne pathogens were tested to demonstrate the functionality of the coatings for food industry applications.

## 2. Materials and Methods

### 2.1. Design of Experiments

The experimental methodology was conducted in two sequential phases. First, a photocatalytically optimized sample was established as the baseline, followed by systematic adjustments to synthetic parameters to identify a local optimum for mechanical performance. Seven distinct coatings were then fabricated by varying sintering temperatures and other synthesis conditions, with their mechanical properties, hardness and elastic modulus evaluated via nanoindentation.

The synthetic parameters for producing the AMCs, including their experimental variation ranges, were selected based on our previous study that optimized the photocatalytic performance of sol–gel TiO_2_ coatings on food-grade stainless steel [25,26,27]. Extreme values of these parameters were tested, encompassing two different synthesis protocols, templating agent-to-precursor (Ti:EO) ratios of 0.5 and 1.2, aging durations of 1 h and 240 h, spin-coating speeds of 2000 and 6000 rpm, and sintering temperatures of 400 °C and 600 °C [25,26,27].

Variations in hardness and elastic modulus were analyzed in accordance with the experimental design outlined in Table 1. Following Lazic’s (2004) methodology [28], this approach facilitated the (1) identification of an optimal parameter combination for improved mechanical properties, and (2) determination of statistically significant factors influencing these properties. Nanoindentation measurements [25,26,27,29] provided the necessary hardness and elastic modulus data, which were then processed using Minitab 18 (Minitab, State College, PA, USA) via its design of experiment (DOE) statistical tools for a full 2-factorial analysis.

Upon identifying sintering temperature as the primary factor affecting mechanical durability, additional experiments were performed to systematically investigate its correlation with coating hardness and elastic modulus. These insights were subsequently applied to develop coatings with enhanced mechanical properties while preserving photocatalytic efficiency.

Protocol 1, adapted from Andersson et al. (2016) [30] for porous anatase formation, involved hydrolyzing 1.0 g titanium(IV) ethoxide (20% Ti in ethanol, Sigma-Aldrich, St. Louis, MO, USA) with 0.8 g fuming HCl (37%, Honeywell Fluka, Morris Plains, NJ, USA) in sealed glass vials. Concurrently, either 0.11 g or 0.25 g of Pluronic P123 (MW ~5800, Sigma-Aldrich) was dissolved in 4.25 g anhydrous ethanol (≥99.5%, Decon Labs, King of Prussia, PA, USA), under magnetic stirring at 1200 rpm, to achieve Ti:EO ratios of 1.2 or 0.5, respectively. The templating solution was then mixed with the hydrolyzed precursor and aged for 1 h or 240 h at a tightly controlled temperature of 25 °C ± 0.1 °C using a temperature-regulated stirring plate (RCT basic with ETS-D5, IKA, Wilmington, NC, USA).

Protocol 2, a modified non-porous anatase synthesis based on Collins (2012) [31], involved a multi-step procedure. Initially, 1.0 g titanium(IV) ethoxide (20% Ti in ethanol, Sigma-Aldrich) was hydrolyzed with 6.4 g deionized water under vigorous stirring (1200 rpm, 5 min) in sealed glass vials. The resulting precipitate was vacuum-filtered, washed repeatedly with DI water, and dried at ambient conditions. The dried powder was then dispersed in 15 g chilled DI water, followed by the dropwise addition of 5.3 g cold hydrogen peroxide (30 wt%, Sigma-Aldrich) while maintaining the reaction vessel in an ice-water bath. The system was periodically vented to release evolved gases until gas formation ceased, after which the sealed vial was stored at 4 °C for 96 h.

Simultaneously, templating solutions were prepared by dissolving either 0.11 g or 0.25 g Pluronic P123 (MW ~5800, Sigma-Aldrich) in 4.25 g anhydrous ethanol (≥99.5%, Decon Labs, USA) under magnetic stirring (1200 rpm). The aged titanium precursor was then brought to room temperature and combined with the templating solution, followed by aging for 1 h or 240 h at 25.0 °C ± 0.1 °C using the RCT basic temperature-regulated stirrer (IKA).

The resulting gels from either protocol were deposited onto stainless-steel substrates using a spin coater (WS-400BZ-6NPP/Lite, Laurell, Lansdale, PA, USA), with relative humidity maintained at 60% via a custom humidification system. Coatings were applied at spin speeds of 2000 rpm or 6000 rpm. Post-deposition, substrates were transferred to a controlled-environment chamber and dried for 24 h at 90% relative humidity.

Sintering was performed using a controlled thermal profile: initial drying at 120 °C, followed by a gradual temperature increase (1 °C/min) to either 400 °C or 600 °C, with a 4 h dwell time at the target temperature.

For the secondary experimental series, coatings were fabricated using Protocol 2 with fixed parameters: a templating agent ratio (Ti:P123) of 1.2, aging duration of 1 h, and spin speed of 2000 rpm. Sintering temperatures were varied across an extended range (Table 2) to enable thorough analysis of temperature-dependent mechanical properties. The resulting data were analyzed using Minitab 18 to establish empirical correlations between sintering temperature and mechanical performance.

### 2.2. Optimized-Coating Fabrication

Upon identifying the synthetic parameters that optimally balanced photocatalytic activity and mechanical durability, final coatings were prepared following Protocol 2 (Section 2.1) under modified conditions: a titanium-to-ethylene oxide ratio of 1.2, aging duration of 1 h, spin-coating speed of 2000 rpm, and sintering temperature of 595 °C. Mirror-finished substrates (No. 8) were used for nanoindentation, while 2B-finished substrates were employed for antimicrobial evaluation, ensuring consistency with prior characterization methods.

### 2.3. Characterization of the Coating

The mechanical and structural properties of all coating variants were systematically evaluated using complementary analytical techniques. For both the initial parameter screening and subsequent trend analysis, coating performance was assessed via nanoindentation-derived hardness and elastic modulus measurements, supplemented by cross-sectional scanning electron microscopy (SEM) thickness analysis. The optimized coatings underwent extended characterization, including thin-film X-ray diffraction (XRD) for crystallographic evaluation, antimicrobial activity assessment, standard mechanical (nanoindentation) and morphological (SEM) profiling.

#### 2.3.1. Structural and Quality Analysis

Optimized coatings were characterized using advanced analytical techniques. Phase identification was conducted via thin-film XRD (Ultima IV, Rigaku, Tokyo, Japan) with Cu K-α radiation (λ = 0.1541 nm) at 40 kV, scanning a 2θ range of 5–80° with a step size of 0.02° and a scan rate of 2°/min. Microstructural evaluation and thickness measurements were performed using field emission SEM (Quanta 600, ThermoFisher Scientific, Waltham, MA, USA) at 30 kV with a 3 nm spot size and minimal aperture setting. Due to the coatings’ inherent conductivity, no additional sample preparation was required.

For precise thickness determination, focused ion beam milling (Scios DualBeam, ThermoFisher Scientific, USA) with 30 kV Ga^+^ ions was used to create clean cross-sections perpendicular to the coating surface. Three-dimensional surface topography was quantified via vertical scanning interferometry (Wyko NT9100, Veeco Instruments, Plainview, NY, USA) using high-resolution white light optical profilometry.

#### 2.3.2. Mechanical Analysis

Mechanical properties were evaluated using a nanoindentation system (G200, Agilent Technologies, Santa Clara, CA, USA) equipped with a calibrated Berkovich diamond indenter (Micro Star Technologies, Huntsville, TX, USA). For each coating, 18 independent indentations were performed under load control up to 200 mN, with continuous stiffness measurements recorded at 10 discrete penetration depths per indentation.

Hardness values were calculated exclusively from data obtained at penetration depths ≤ 10% of the total coating thickness, adhering to established protocols to minimize substrate interference [32,33,34]. The reduced elastic modulus (E_r_) was determined through unloading curve analysis using the Oliver–Pharr method.

#### 2.3.3. Antimicrobial Activity

##### Coating Effects on Attached Bacteria

The optimized coating’s antimicrobial activity was assessed by exposing it to two pathogenic bacterial strains, *Escherichia coli* O157:H7 strain 505B and *Staphylococcus aureus* strain FRI, from the University of Missouri Food Microbiology Lab culture collection and determining bacterial reduction via agar plating methods. Two sterilized coating samples and one sterilized uncoated stainless-steel substrate were placed inside a sterile plastic Petri dish lined with a sterile moist Whatman filter paper (No 4), as shown in Supplementary Figure S2. Samples were inoculated by evenly spreading 100 µL of freshly grown bacterial culture (10^8^ CFU/mL), followed by air-drying until completely dried (~40 min). The control substrate and the photocatalytic coatings (named sample 1 and sample 2, respectively) were placed facing upwards on a sterile glass slide (to avoid direct contact with the moist paper). The Petri dish was covered with its lid, and a 365 nm UV-A lamp (365 nm, 6 Watts, UVP UVL-56, Analytikjena, Upland, CA, USA) was turned on and placed over the Petri dish, 10 cm above the coatings’ surfaces, as shown in Supplementary Figure S2. The entire set was incubated at 37 °C for different periods of time, *viz*., 0, 3, 6, 12, and 24 h. Another set of samples was identically prepared but incubated in the dark at 37 °C, covering the entire Petri dishes with aluminum foil to protect the samples from light. At each sampling time, samples were suspended in 10 mL sterile peptone water and vortexed on high for 2 min to detach the bacteria from the samples and to re-disperse them in solution. The bacterial solutions were serially diluted and pour-plated in Tryptic Soy agar (TSA) (Bacto, Becton, Dickinson and Co., Franklin Lakes, NJ, USA). Plates were incubated for 24 h at 37 °C, and bacterial counts were recorded as CFU/mL.

##### Coating Effects on Bacterial Biofilms

The effects of the coatings on *E. coli* O157:H7 505B and *S. aureus* FRI biofilms were also assessed. Each strain was separately pre-enriched in TSB for 20 h at 37 °C. Autoclaved coatings and bare stainless-steel substrates with a 2B finishing were placed in individual wells of a sterile 6-well microplate. Each freshly grown bacterial suspension (1 mL) was added to individual wells in triplicate, and 2 mL of TSB was added to each well to ensure that the bacterial suspension completely covered the surface of the coupons. The TSB was refreshed every 12 h to remove planktonic cells in the suspensions. Plates were incubated for 48 h at 37 °C, and unattached cells on both sides of the coupons were gently removed by rinsing with sterile 1 mL peptone water using a micropipette. All coupons were gently transferred to glass slides in Petri dishes lined with Whatman filter paper moistened with 5 mL DI water to prevent drying out during incubation. UV-A treatment and dark treatment were separately conducted, as described above (Figure S2), and sampling was performed at 0 h, 3 h, 6 h, 12 h and 24 h. For sampling, 1 mL sterile peptone water was pipetted over each coupon four times to gently rinse off loosely attached cells. Ultrasonication (5 min, 120 V, 50 kHz) was then performed to dislodge all biofilm structures from the stainless-steel coupons. Then, each coupon was vortexed at medium speed for 2 min. Bacterial suspensions were serially diluted in peptone water and pour-plated in TSA in duplicate. Plates were incubated for 24 h at 37 °C.

## 3. Results

### 3.1. Synthetic Factors Screening Experiment

#### 3.1.1. Microstructure and Crystal Phase

**Figure 1:**
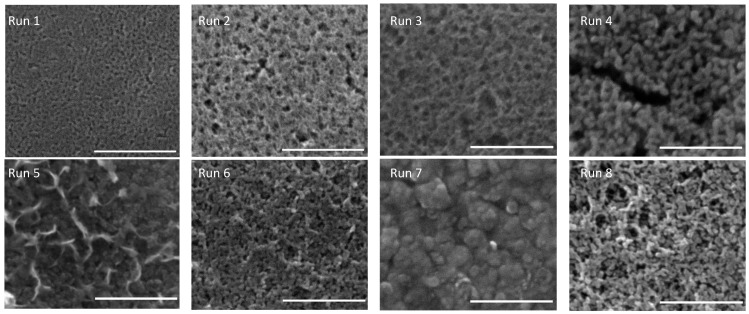
Representative coatings’ microstructure after treatments corresponding to the eight different runs of the screening of factors experiment. All scale bars are 200 nm [25,27].

Figure 1 presents characteristic microstructural features of TiO_2_ coatings fabricated under diverse processing conditions (as specified in Table 1) derived from synthetic factor screening experiments. The observed variations in porosity demonstrate that extended gel aging durations and elevated sintering temperatures facilitate the densification of porous architectures, resulting in the development of TiO_2_ aggregates. Of particular relevance are the microstructural characteristics of Run 2 and Run 7 coatings, which merit special attention due to their exceptional hardness values under Protocol 1 and Protocol 2, respectively.

**Figure 2:**
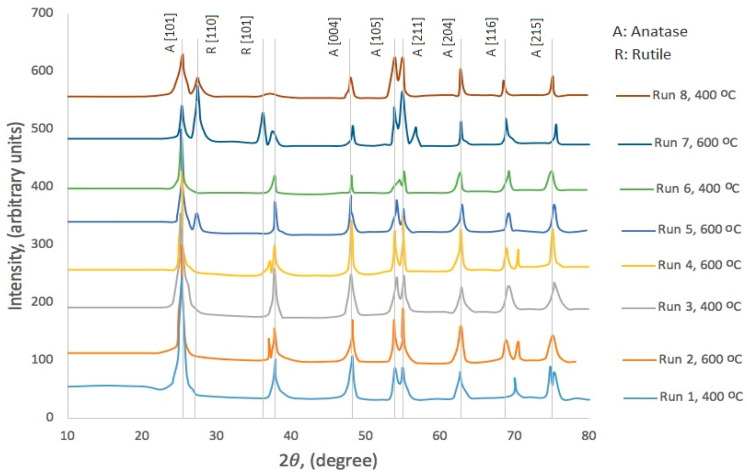
X-ray diffraction patterns comparison between coatings from the eight runs’ treatments. The patterns follow trends that can be related to the type of protocol and the sintering temperature used to prepare them [25,27].

Thin-film XRD analysis of the crystalline phases produced under eight distinct treatment conditions (Table 1) revealed phase composition dependencies on synthesis parameters (Figure 2). The coatings comprised either pure anatase or mixed anatase-rutile phases, with Protocol 1 (Runs 1–4) predominantly yielding anatase and Protocol 2 (Runs 5–8) generating mixed-phase systems. The emergence of a minor rutile [101] reflection in Protocol 1 coatings sintered at 600 °C, absent at 400 °C, demonstrates the temperature-dependent phase transformation. A parallel phenomenon occurs in Protocol 2 coatings, where the rutile [110] peak attenuates at 400 °C (disappearing completely in Run 6) but intensifies at higher temperatures (becoming dominant in Run 7).

Notably, diffraction pattern variations persist among coatings processed under identical protocols and temperatures. For instance, the anatase [215] reflection differs between Run 1 and Run 3 (both sintered at 400 °C), while anatase ([101], [004], [211]) and rutile ([110], [101]) peak intensities vary between Run 5 and Run 7 (both sintered at 600 °C). These inconsistencies imply that additional parameters, including gel aging duration and precursor composition, influence phase evolution and crystallinity. These inconsistencies imply that additional parameters, including gel aging duration and precursor composition, influence phase evolution and crystallinity.

Crystallite dimensions, calculated from the [101] peak’s FWHM using the Scherrer equation (Table 1), exhibit dependence on synthesis protocol, sintering temperature, and surfactant concentration. Protocol 2 consistently produced larger crystallites than Protocol 1, attributable to fundamental differences in sol–gel chemistry: Protocol 1’s acid-catalyzed conditions (pH < 2, low H_2_O:Ti ratio) yield weakly branched linear polymers, while Protocol 2’s peroxo-catalyzed system (pH > 2, high H_2_O:Ti ratio) forms densely branched compact networks. These structural distinctions manifest during thermal processing, *viz*., weakly branched precursors favor densification with limited crystallite growth, whereas highly branched systems promote simultaneous sintering and crystallite coarsening [35]. Furthermore, increased Ti/surfactant ratios (1.2 vs. 0.5) coupled with 600 °C sintering enhanced crystallite enlargement. As reported in [36], 1.2:1 precursor/surfactant molar ratios generate cubic nanostructures with concave pore architectures. At elevated temperatures, accelerated material redistribution into these pores drives both pore collapse and crystallite growth [36].

#### 3.1.2. Nanoindentation Hardness and Elastic Modulus

**Figure 3:**
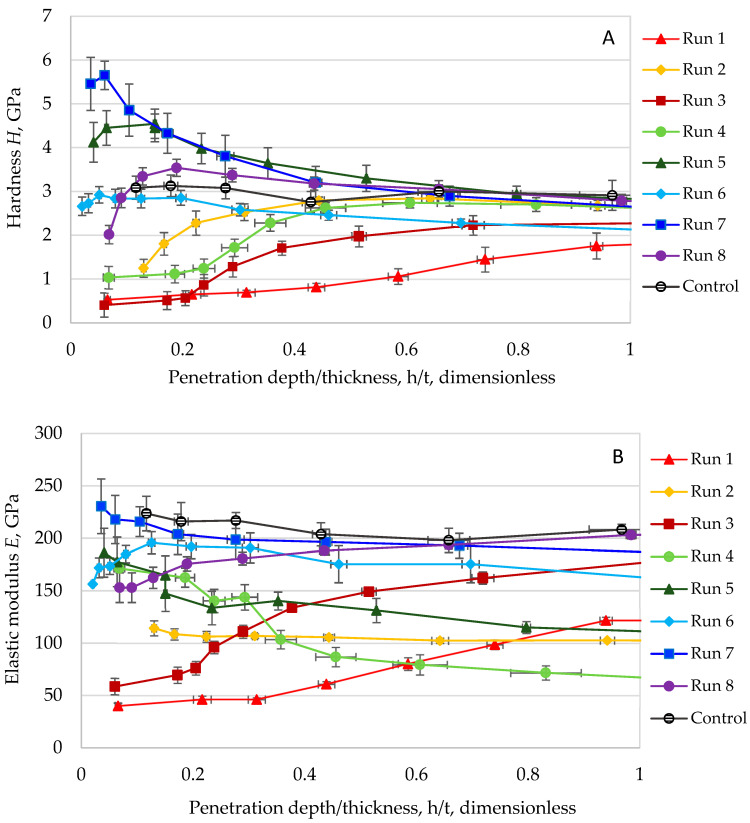
Overall hardness values (**A**) and nanoindentation elastic moduli at different displacement ratios (*h*/*t*) (**B**) from the screening of synthetic factors experiment. The displacement ratio (*h*/*t*) was used as a basis for comparison between samples [25,27].

The mechanical behavior of the synthesized TiO_2_ coatings was evaluated through nanoindentation analysis, with Figure 3A and Figure 3B presenting the hardness–displacement and elastic modulus–displacement ratio curves, respectively, for all eight coating variants, as defined by Equation (1). Both mechanical properties demonstrated significant dependence on *contact penetration depth*, necessitating normalization through the displacement ratio (*h_c_*/*t*) to enable direct comparison across samples with varying thicknesses, a standard practice in thin-film characterization [32].

The observed depth-dependent mechanical response originates from the progressive development of strain fields during indentation. At minimal penetration depths, *h_c_*, the measured properties reflect the intrinsic characteristics of the coating material. As indentation progresses, the expanding strain field increasingly incorporates contributions from the substrate, resulting in a composite mechanical response that blends both coating and substrate behaviors.

(1) $$h=\frac{contact\;penetration\;depth,\;nm}{coating\;thickness,\;nm}=(h_{c}/t)$$

To accurately represent these measurements, the terms effective hardness and effective modulus were adopted, acknowledging the composite nature of the data while accounting for the substantial porosity inherent to these coatings, as detailed in Section 3.4.2. This terminology explicitly recognizes that the measured properties represent an integrated response from the porous TiO_2_ matrix rather than solely the dense material phase.

Comparative analysis revealed systematic differences between coating protocols. Coatings produced via Protocol 1 consistently demonstrated lower effective hardness values than their Protocol 2 counterparts. All hardness profiles asymptomatically approached the substrate baseline with increasing indentation depth, establishing a critical measurement constraint. Data acquired beyond 10% of the *coating thickness* became progressively influenced by substrate effects [32], prompting restriction of quantitative analysis to the *h*_c_/*t* ≤ 0.1 regime to ensure coating-dominated responses. The analysis yielded several important findings. Protocol 2 uniformly produced coatings with enhanced mechanical resistance, although the hardness disparity between protocols diminished at shallow penetration depths, suggesting potential divergence between surface and bulk properties. Run 7 emerged as the hardest coating, while Run 3 exhibited the lowest hardness, a particularly noteworthy contrast given their crystallite size relationship. As discussed in Section 3.1.1, Run 7 featured marginally larger crystallites than Run 3, yet demonstrated an order-of-magnitude greater hardness. Considering Equation (2), an explanation for the difference in the observed hardness values could be given:

(2) $$\sigma =kG^{-1/2}$$

where *σ* is the strength (yield stress) (Pa), *k* is the material-specific Hall–Petch constant (Pa m^1/2^), and *G* is the grain size (m).

For ceramic materials, tensile strength exhibits an approximate linear correlation with indentation hardness [19]. As expressed in Equation (1), this relationship predicts that reduced grain size typically enhances hardness. While grain size was not explicitly characterized in this study (where a grain may comprise single or multiple crystallites), the observed crystallite size data reveal an apparent anomaly: Run 3 exhibited marginally larger crystallites than Run 7 yet demonstrated a tenfold lower hardness.

This apparent contradiction to conventional Hall–Petch behavior underscores the dominant role of microstructural porosity over crystallite size in these systems. The substantial porosity difference between Run 3 (38%) and Run 7 (0%) fundamentally altered their mechanical response, demonstrating that porosity outweighs crystallite size effects in governing hardness for these TiO_2_ coatings. The results further reveal that microstructural integrity dictates mechanical performance, with dense coatings achieving superior hardness despite slightly larger crystallites, while simultaneously influencing both photocatalytic activity and mechanical robustness through dual structure–property relationships.

Parallel trends emerged in elastic modulus measurements, with Protocol 2 coatings again outperforming Protocol 1 specimens. While modulus values showed less distinct convergence toward the substrate baseline compared to hardness, the relative ranking remained consistent, with Run 7 exhibiting the highest modulus and Run 1 the lowest. Intermediate samples followed the same hierarchy observed in hardness testing.

The nanoindentation characterization faced multiple challenges, including inherent material brittleness, high porosity, sub-micron thickness effects, and increasing substrate influence at greater penetration depths. To ensure data validity, analysis was strictly limited to the regime where coating properties dominate. While absolute values require cautious interpretation due to these constraints, the dataset enables reliable comparative assessment across coatings.

Table 1 summarizes average hardness and modulus values derived from the appropriate measurement regime, revealing consistent trends. Protocol 2 coatings demonstrated superior mechanical performance, with Run 7 exhibiting significantly greater stiffness than Run 1. The modulus trends mirrored hardness behavior, reinforcing their mechanistic linkage, while the results align with established structure–property relationships for oxide coatings where density governs mechanical performance [25,26,27,29].

### 3.2. Trends Determination Experiment

Response surface analysis (Lazic [28]) applied to the mechanical properties data (Table 1) established protocol type and sintering temperature as statistically significant factors. Given its demonstrated mechanical superiority, Protocol 2 was chosen for more detailed investigation. Subsequent experiments systematically varied sintering temperature according to Lazic’s [28] rotatable design while holding other parameters constant, including aging time (48 h), spinning speed (3000 rpm), and surfactant ratio (0.5 Ti:EO), as outlined in Table 2.

**Figure 4:**
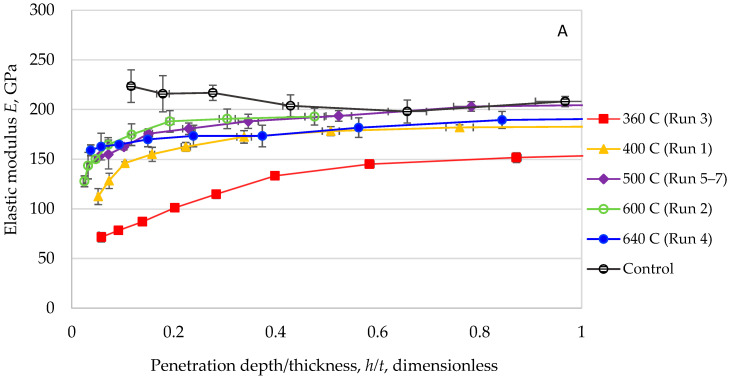

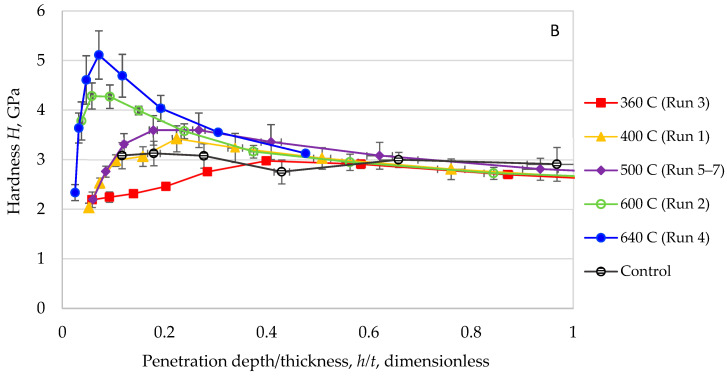
Trend determination experiment showing increasing coatings’ elastic modulus values (**A**) and hardness values (**B**) with increasing sintering temperature [25,27].

The evolution of elastic modulus across different displacement ratios for coatings sintered between 360 and 640 °C is presented in Figure 4A. Several consistent patterns were observed. Modulus values exhibited a monotonic increase with rising sintering temperature, though all coatings remained less stiff than the stainless-steel substrate (~200 GPa). Notably, samples processed at higher temperatures (≥600 °C) approached but did not exceed 75% of the substrate’s stiffness.

A parallel relationship emerged in hardness measurements, as shown in Figure 4B. The enhancement of hardness strongly correlated with thermal treatment intensity. As previously discussed in Section 3.1, elevated temperatures promoted both grain growth and porosity reduction, resulting in denser microstructures. The maximum hardness values (~4 GPa at 595 °C) reached approximately 80% of the substrate’s intrinsic hardness.

**Figure 5:**
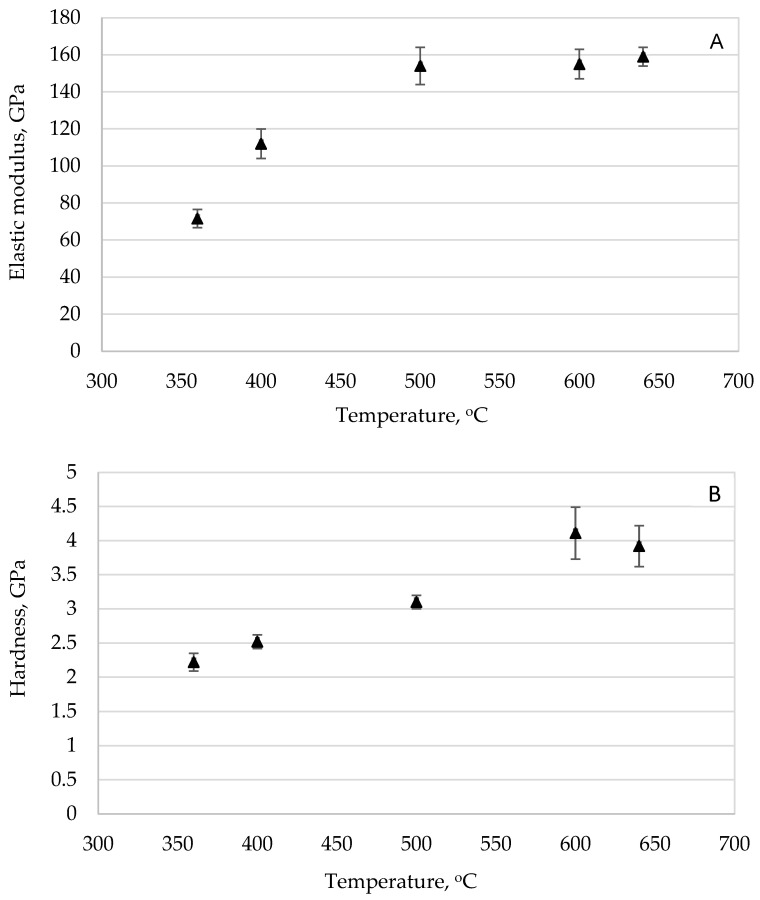
For the trend determination experiments, the coatings’ hardness (**A**) and elastic modulus (**B**) values increased as sintering temperature increased [25,27].

These relationships are quantitatively summarized in Figure 5, demonstrating consistent improvements in both modulus and hardness across the experimental temperature range. The optimal performance conditions were achieved at different temperatures—maximum modulus (~150 GPa) occurred at 640 °C, while peak hardness (~4 GPa) was observed at 595 °C. This divergence suggests distinct structural dependencies: elastic modulus appears more strongly influenced by complete pore elimination, while hardness may benefit from preserving nanocrystalline structure before excessive grain growth occurs.

The temperature-dependent behavior underscores the complex interplay between microstructural evolution and mechanical properties in these coating systems. Higher sintering temperatures generally improved mechanical performance through densification and structural refinement, although the specific optimal conditions varied between modulus and hardness measurements. This difference highlights the need for balanced processing conditions when designing coatings for applications requiring both stiffness and hardness.

### 3.3. Optimization of Hardness, Elastic Modulus, and Photocatalytic Activity

**Figure 6:**
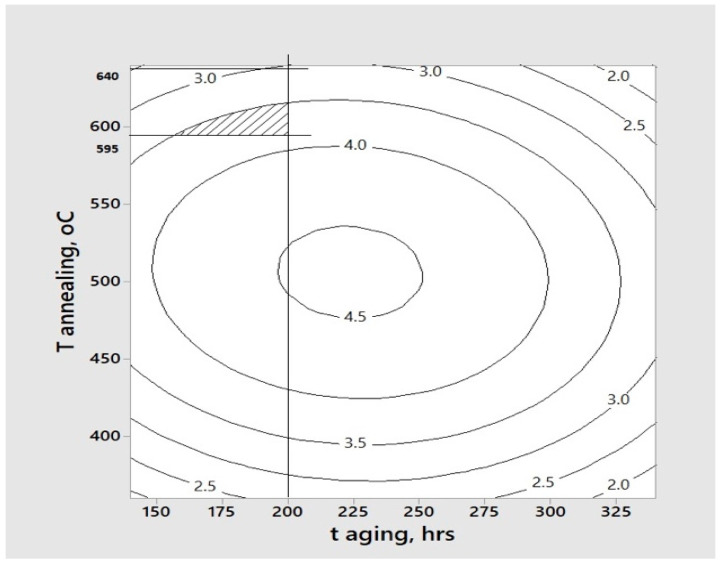
The contour plot of the fading speed (a descriptor of photocatalytic activity) as a function of aging time and sintering temperature was used to locate the sintering temperature and the aging time needed to fabricate a coating with balanced values of fading speed, elastic modulus, and hardness. The optimal region is highlighted by the dashed area [25,27].

By combining our previously established mathematical model for photocatalytic activity [26] with the mechanical property trends from Section 3.2, Figure 6 was developed to identify the optimal sintering temperature that balances photocatalytic efficiency, hardness, and elastic modulus. This comprehensive visualization integrates three critical datasets: (1) the photocatalytic activity response surface, (2) the maximum hardness observed at 595 °C, and (3) the elastic modulus peak at 640 °C.

The analysis uncovered a fundamental materials optimization challenge across the investigated temperature range (360–640 °C). Photocatalytic activity reached its maximum at 525 °C before decreasing with further heating, while mechanical properties exhibited contrasting behavior. Hardness showed continuous improvement with increasing temperature, and elastic modulus approached its maximum near 600 °C. This inverse relationship between functional and mechanical performance required a carefully considered compromise in our optimization strategy, where photocatalytic activity was most heavily prioritized, followed by hardness, with elastic modulus receiving relatively lower consideration.

These temperature-dependent trade-offs originate from fundamental material phenomena. Elevated sintering temperatures enhance density and mechanical properties through pore elimination and grain growth but simultaneously diminish photocatalytic activity by reducing surface area and modifying phase composition. This intrinsic interdependence highlights the critical need for application-specific parameter selection when engineering photocatalytic coatings that must maintain mechanical durability.

Photocatalytic activity was accorded the highest priority in our optimization scheme as it directly governs the coatings’ antimicrobial properties, their primary functional characteristic. Hardness was selected as the secondary priority over elastic modulus due to its greater practical significance in resisting localized plastic deformation when the coatings are subjected to external stressors during cleaning and sanitation procedures of FCSs. These stresses may include various mechanical cleaning methods, such as blasting, ice pigging, and scrubbing [6].

The optimization process revealed that the ideal sintering temperature represents a careful balance between competing material properties, where maximizing one characteristic often comes at the expense of another. This finding emphasizes the complex nature of multifunctional coating design and the importance of establishing clear performance hierarchies based on intended application requirements. The methodology developed here provides a framework for tailoring coating properties to specific operational needs while maintaining acceptable levels of all critical performance characteristics.

### 3.4. Optimized Coating’s Structural and Quality Analysis

#### 3.4.1. X-Ray Diffraction (XRD)

**Figure 7:**
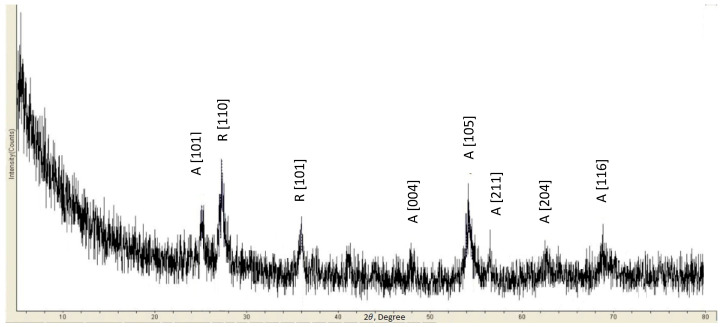
The optimized coating’s XRD pattern showing anatase and rutile phases, the latter giving higher intensity peaks [25,27].

The XRD analysis of the optimized coating (Figure 7) verified the simultaneous presence of both anatase and rutile TiO_2_ polymorphs, with rutile demonstrating substantially greater diffraction intensity. Quantitative phase analysis indicated a rutile predominance of 60%, reflecting the synthesis conditions that emphasized mechanical performance. This specific phase distribution carries considerable importance as it directly governs two essential coating characteristics: photocatalytic functionality and mechanical durability.

The measured phase composition corresponds with well-documented structure–property relationships in TiO_2_ systems. The prevalence of rutile phase results from the thermal optimization process, where elevated sintering temperatures promoted formation of the thermodynamically stable rutile polymorph while concurrently improving mechanical properties through microstructural densification. This phase evolution, however, presents a fundamental trade-off, as the enhanced mechanical characteristics come at the cost of reduced photocatalytic efficiency, given rutile’s typically lower activity compared to anatase.

Notably, the retained 40% anatase content preserves adequate photocatalytic functionality, demonstrating that our optimization methodology effectively reconciled these competing material requirements. The successful retention of the anatase phase, despite the high-temperature processing conditions, suggests careful control of thermal parameters during sintering, achieving sufficient phase stability to maintain photocatalytic activity while still obtaining the mechanical benefits associated with rutile formation and densification.

#### 3.4.2. Scanning Electron Microscopy

**Figure 8:**
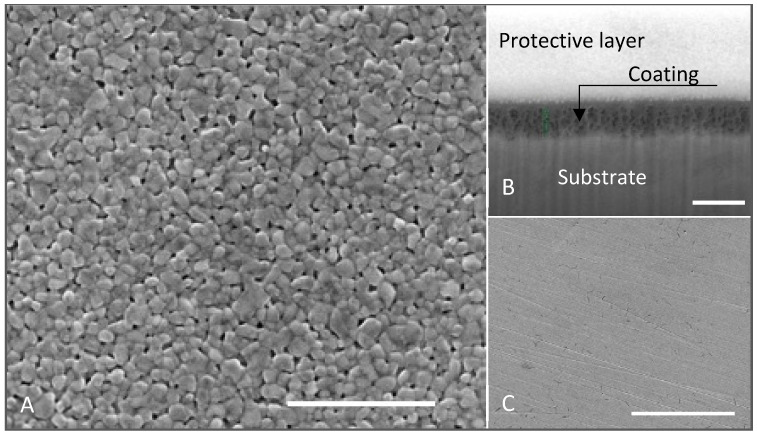
SEM micrographs of optimized coating structure: nanostructure (**A**), scale bar is 500 nm; 235 nm coating thickness (**B**), scale bar is 400 nm; and macroscopic coating’s surface (**C**), scale bar is 100 µm [25,27].

Figure 8 presents representative micrographs at varying magnifications, revealing the characteristic microstructural features of the optimized coating. At the nanoscale level, the images display sintered, flattened particles with rounded morphology arranged in a close-packed configuration. Top-view imaging clearly revealed numerous pores distributed throughout the surface, along with the formation of interparticle necks, morphological features indicating the concurrent occurrence of both coarsening (neck growth without particle movement) and sintering (particle rearrangement) processes during thermal treatment.

The application of combined SEM and ion beam milling techniques enabled cross-sectional examination, demonstrating that the observed surface porosity extended throughout the coating thickness. The coating exhibited remarkably few macroscopic defects, with the exception of localized cracking patterns that followed the striation lines inherent to the industrial-grade stainless-steel substrate. This minimal defect formation can be explained by two key factors: first, the presence of an interconnected porous network within the coating structure facilitated stress dissipation during drying by accommodating capillary pressure-induced stresses; second, the controlled coating thickness of approximately 235 nm minimized solvent concentration gradients between the coating’s inner layers and the surrounding environment during the spin-coating and drying stages [36].

#### 3.4.3. Hardness and Elastic Modulus

The confirmatory experiment performed as the final stage of the response surface optimization process yielded a strong correlation between predicted and experimentally determined mechanical properties of the optimized coating. Hardness measurements of 3.8 ± 0.3 GPa closely approximated the model’s prediction of 4.2 ± 0.3 GPa based on the trends identified in Section 3.2. Elastic modulus values showed comparable agreement, with experimental results of 125 ± 20 GPa aligning reasonably with the anticipated 155 ± 5 GPa, particularly when accounting for the characteristic variability associated with thin film mechanical characterization. These findings substantiate sintering temperature as the primary determinant of mechanical properties within the studied thermal processing range.

**Figure 9:**
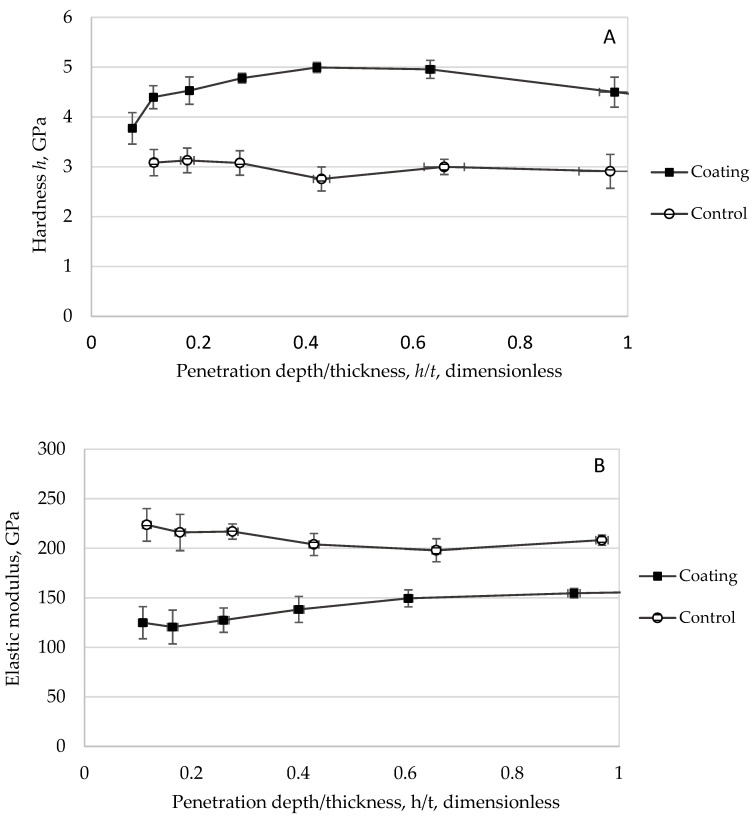
The optimized coating’s hardness (**A**) observed was 4.0 GPa, while the elastic modulus (**B**) observed was 125 GPa, fairly close to what was expected from the trends obtained during the optimization process. The control’s values correspond to bare stainless [25,27].

Figure 9 provides additional insight into the temperature-dependent mechanical behavior, plotting both hardness and elastic modulus as functions of sintering temperature. In accordance with the standardized methodology implemented throughout this investigation, all reported mechanical properties were measured at penetration depth-to-thickness ratios ≤ 0.1 to guarantee characterization of intrinsic coating properties while minimizing substrate effects. The data clearly demonstrate the progressive convergence of both mechanical parameters toward bulk stainless-steel substrate values with increasing indentation depth, a consistent observation across all experimental conditions that emphasizes the critical need for controlled penetration depths when evaluating coating-specific mechanical performance.

The observed minor variations between predicted and experimental values likely originate from several intrinsic aspects of the coating process. Potential contributing factors include localized porosity distribution fluctuations, subtle variations in phase composition, and nanoscale microstructural heterogeneity—all inherent characteristics of sol–gel derived coatings that may lead to deviations from idealized model projections. Despite these minor discrepancies, the strong overall agreement between theoretical predictions and empirical measurements confirms the robustness of the response surface methodology for optimizing these multifunctional photocatalytic coatings.

### 3.5. Coating Effects on Pathogenic Bacteria

#### 3.5.1. Effects of Protocol 1 and 2 Coatings on Attached Cells

**Figure 10:**
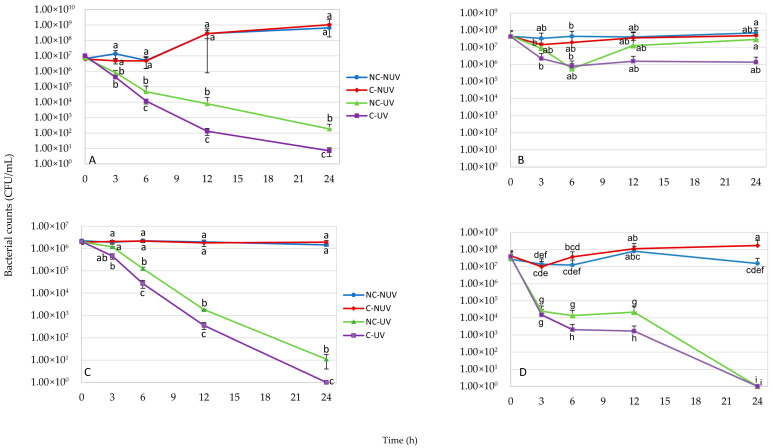
Antimicrobial effect of the antimicrobial coatings on attached *E. coli* O157:H7 ((**A**) [25,27], (**B**)) and *S. aureus* (**C**,**D**). Coatings were produced using optimized Protocol 1 (**A**,**C**) and Protocol 2 (**B**,**D**). C-UV (coating with UV light), NC-UV (no coating with UV light), C-NUV (coating with no UV light), and NC-NUV (no coating with no UV light). Error bars indicate standard deviations from three measurements. Different lowercase letters on each experimental point indicate significant differences (*p* ≤ 0.05).

Figure 10 presents the survival rates of Gram-negative *E. coli* O157:H7 505B (A,B) and Gram-positive *S. aureus* FRI (C,D) following exposure to coatings prepared via Protocol 1 and Protocol 2, over increasing incubation periods. As anticipated, neither the C-NUV nor the NC-NUV treatment inhibited either bacterial strain, regardless of the coating protocol employed. No significant differences (*p* > 0.05) in bacterial viability between these treatments were observed at any time point, confirming the absence of inherent antibacterial activity in the optimized coatings under dark conditions.

In contrast, the C-UV and NC-UV treatments demonstrated significant reductions (*p* ≤ 0.05) of both strains beginning at 3 h, when compared to the C-NUV and NC-NUV treatments in Protocol 1 coatings. Notably, coatings produced via Protocol 2 did not exhibit a statistically significant decline in *E. coli* O157:H7 populations over the exposure duration. Long-wave UV irradiation exerted a progressive bactericidal effect, as evidenced by the descending trend in the NC-UV curves for both bacteria. This aligns with the established use of UV light as a mild antimicrobial intervention for food contact surfaces, owing to its capacity to damage microbial DNA.

Examination of the C-UV curves for each bacterial strain revealed enhanced antimicrobial efficacy, with the data points consistently positioned below all other treatment curves, particularly beneath the NC-UV trajectory. This observation confirms not only the combined antimicrobial effect of the treatment but also the intrinsic antibacterial properties of the coating itself. Specifically, Protocol 1 coatings subjected to 24 h of UV exposure (C-UV) achieved approximately 7.5-log and 6.5-log reductions in *E. coli* O157:H7 and *S. aureus* FRI, respectively, relative to the NC-NUV and C-NUV controls. Furthermore, after 12 h, the C-UV treatment yielded an additional 1.0-log reduction compared to the NC-UV treatment, indicating a synergistic interaction between the coating and UV light.

To isolate the coating’s specific contribution to bacterial inactivation, counts from NC-UV treatments were subtracted from those of C-UV. Given that real-world applications necessitate combined coating-UV deployment, the total reduction of approximately 10^7^ CFU/mL underscores the potential of this approach for controlling bacterial contamination on stainless-steel FCSs.

The time-dependent inactivation kinetics depicted in Figure 10 also suggest practical utility in industrial food processing environments, particularly for batch operations with standard 8 h production cycles. During such intervals, one production line can remain operational while another undergoes concurrent UV-coating sanitation. After 8 h of UV exposure, Protocol 1 coatings reduced bacterial loads by roughly 1.0 × 10^3^ CFU/mL for both test organisms, while Protocol 2 coatings achieved comparable reductions only for *S. aureus*, as illustrated in Figure 10A,C,D.

All data were derived from triplicate experiments and are presented as mean values ± standard deviations. Statistical significance was assessed via one-way ANOVA followed by Tukey’s post hoc test to differentiate treatment means.

#### 3.5.2. Effects of Protocol 2 Coatings on Pathogenic Biofilms

**Figure 11:**
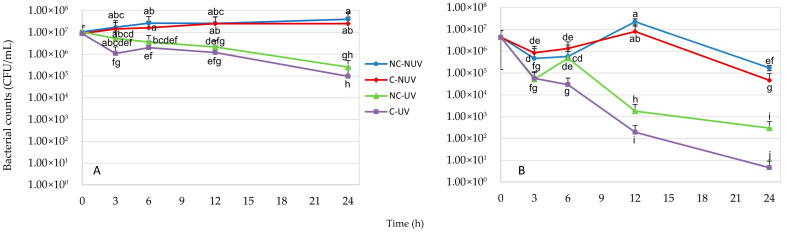
Antimicrobial effect of the antimicrobial coatings on *E. coli* O157:H7 (**A**) and *S. aureus* (**B**) biofilms. Coatings were produced using optimized Protocol 2. The results shown in the plot correspond to the following treatments: C-UV (coating with UV light), NC-UV (no coating with UV light), C-NUV (coating with no UV light), and NC-NUV (no coating with no UV light). Error bars indicate standard deviations from three measurements. Different lowercase letters on each experimental point indicate significant differences (*p* ≤ 0.05).

After 3 h of treatment, the combination of UV irradiation and Protocol 2 coating achieved a reduction exceeding 1 log CFU/mL in *E. coli* O157:H7 biofilm counts compared to non-UV-treated groups, along with a 0.8 log reduction relative to UV-treated, uncoated surfaces (*p* ≤ 0.05). However, no further statistically significant reductions (*p* > 0.05) were observed in either coated or uncoated groups subjected to UV treatment at later time points (Figure 11A). In contrast, a 1 log CFU reduction in *S. aureus* biofilm was observed after 6 h of UV exposure compared to uncoated controls (*p* ≤ 0.05) (Figure 11B). Extending the treatment duration to 12 h resulted in a pronounced 5 log CFU decrease in *S. aureus* biofilm when treated with Protocol 2 coating and UV light, relative to untreated controls, and a 1 log CFU reduction compared to UV treatment alone (*p* ≤ 0.05).

The observed variations in antibacterial efficacy were influenced by multiple factors, including the coating protocol employed, bacterial classification (Gram-negative versus Gram-positive), cellular morphology (rod-shaped versus spherical), cell wall structure, and growth state (planktonic or biofilm). Notably, the Protocol 2 coating demonstrated negligible activity against *E. coli* O157:H7, a Gram-negative, rod-shaped bacterium, irrespective of its planktonic or biofilm state. In contrast, both Protocol 1 and Protocol 2 coatings exhibited substantial antibacterial effects against *S. aureus*, a Gram-positive, spherical bacterium, whether in planktonic or biofilm form. These findings suggest that the antimicrobial performance of the coatings is highly dependent on the structural and physiological characteristics of the target microorganisms.

## 4. Conclusions

This study employed a sequential response surface methodology incorporating synthetic factor screening and empirical model development, complemented by an overlaying contour plot analysis, to optimize the balance between photocatalytic activity, hardness, and elastic modulus in a nanostructured antimicrobial coating. The optimal fabrication parameters were determined to be a peroxo-catalyzed sol–gel-derived gel aged for 200 h, spin-coated at 2000 rpm, and sintered at 595 °C. These conditions yielded a coating with exceptional photocatalytic performance, mechanical durability, and antibacterial properties, positioning it as a promising sanitation strategy for food contact surfaces (FCS). This approach could be synergistically integrated with existing sanitation protocols, particularly in time-sensitive industrial processes, where rapid and effective microbial control is paramount.

The antibacterial and antibiofilm performance of the coatings exhibited species- and treatment-dependent variability. Specifically, the Protocol 1 TiO_2_ coating demonstrated significant growth inhibition against both Gram-positive and Gram-negative bacteria within six hours of application. Meanwhile, the Protocol 2 TiO_2_ coating showed pronounced efficacy against Gram-positive *S. aureus*, effectively suppressing both planktonic cells and biofilms after six hours of treatment. These findings underscore the tailored applicability of each coating protocol based on microbial targets and operational requirements in industrial settings.

## Figures and Tables

**Table 1:** Full, 2-factorial design of experiment used for screening the significant synthetic factors, in order to identify those that affect the hardness and elastic modulus of the resulting TiO_2_ coatings.

Run	Synthetic Factor					Coating Thickness, nm	Response		Crystallite Size, nm
	Protocol	Ti:EO ^1^	t_aging_, h	rpm	T_sintering_, °C		Hardness, GPa	Elastic Modulus, GPa	
1	1	1.2	1	6000	400	608 ± 33	0.527 ± 0.02	39.9 ± 2.8	15.54
2	1	1.2	1	2000	600	688 ± 51	1.28 ± 0.21	118 ± 7	18.32
3	1	0.5	240	6000	400	905 ± 82	0.394 ± 0.18	60.6 ± 7.9	18.32
4	1	0.5	240	2000	600	1103 ± 94	1.03 ± 0.26	171 ± 3	16.70
5	2	1.2	240	6000	600	1156 ± 122	4.27 ± 0.45	188 ± 23	24.15
6	2	1.2	240	2000	400	1750 ± 198	2.66 ± 0.21	168 ± 9	17.71
7	2	0.5	1	6000	600	825 ± 76	5.32 ± 0.61	221 ± 26	19.95
8	2	0.5	1	2000	400	854 ± 84	2.48 ± 0.21	153 ± 14	22.68

^1^ Surfactant micelle diagrams are expressed in terms of ratios (Ti:EO) of titanium atoms in solution to ethylene oxide groups in solution; therefore, this parameter was chosen as a synthetic factor [25,27].

**Table 2:** A second-order, rotatable design of experiments with the sintering temperature of the AMCs as the single factor affecting hardness and elastic modulus (the experimental responses).

Run	Synthetic Factor						Response	
	Protocol	Ti:EO ^1^	t_aging_, h	rpm	T_sintering_, °C	Coating Thickness, nm	Hardness, GPa	Elastic Modulus, GPa
1	2	1.2	1	2000	400	988 ± 11	2.52 ± 0.10	112 ± 8
2	2	1.2	1	2000	600	1005 ± 8	4.11 ± 0.38	155 ± 8
3	2	1.2	1	2000	360	1102 ± 10	2.22 ± 0.13	72 ± 5
4	2	1.2	1	2000	640	1021 ± 13	3.92 ± 0.30	159 ± 5
5	2	1.2	1	2000	500	895 ± 18	3.10 ± 0.08	154 ± 15
6	2	1.2	1	2000	500	1054 ± 14	3.16 ± 0.10	144 ± 10
7	2	1.2	1	2000	500	1073 ± 12	2.77 ± 0.06	153 ± 12

^1^ Surfactant micelle diagrams are expressed in terms of ratios (Ti:EO) of titanium atoms in solution to ethylene oxide groups in solution; therefore, this parameter was chosen as a synthetic factor [25,27].

## Data Availability

The original contributions presented in this study are included in the article/Supplementary Material. Further inquiries can be directed to the corresponding authors.

## Supplementary Materials

The following supporting information can be downloaded at https://www.mdpi.com/article/10.3390/ma18245640/s1. Figure S1: Surface roughness profiles of the eight coatings prepared for the screening of factors experiments; Figure S2: A schematic of the antimicrobial testing environment; Figure S3: Scanning electron micrographs of typical indents made on samples prepared for the screening of factors experiment; Figure S4: Pareto chart from the screening of factors experiment having coatings’ hardness as the experimental response; Figure S5: Pareto chart from the screening of factors experiment having coatings’ elastic modulus as the experimental response.
