# Supplementary material for: A Photocatalytic TiO2 Coating with Optimized Mechanical Properties Shows Strong Antimicrobial Activity Against Foodborne Pathogens
Source: Materials (Basel). 2025 Dec 15;18(24):5640. doi: 10.3390/ma18245640 (PMC12734942; doi:10.3390/ma18245640)
Supplement: Supplementary file 1 [file materials-18-05640-s001.zip › materials-3469422-supplementary.pdf]

# Supplementary information

## Sink-in and roughness verification

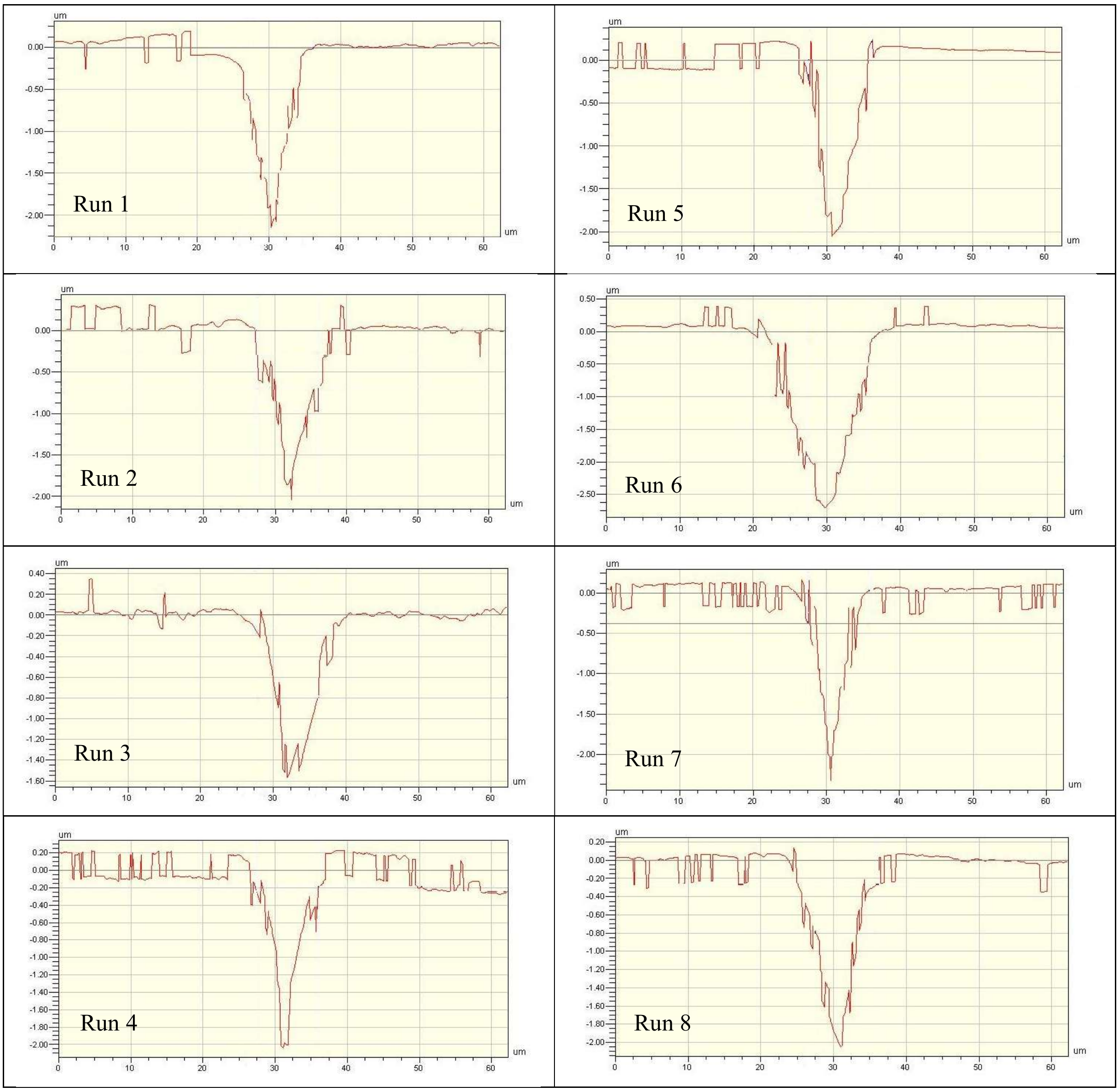


Figure S1. Surface roughness profiles of the eight coatings prepared for the screening of factors experiments. The profiles showed that piling up was absent around the indents, with the exception of Run 4. It can also be observed that the coatings' roughness is negligible in comparison to indent size. *y*-*x* axes are lengths in micrometers [Torres Dominguez, E. 2020. Ph.D. Dissertation, University of Missouri].

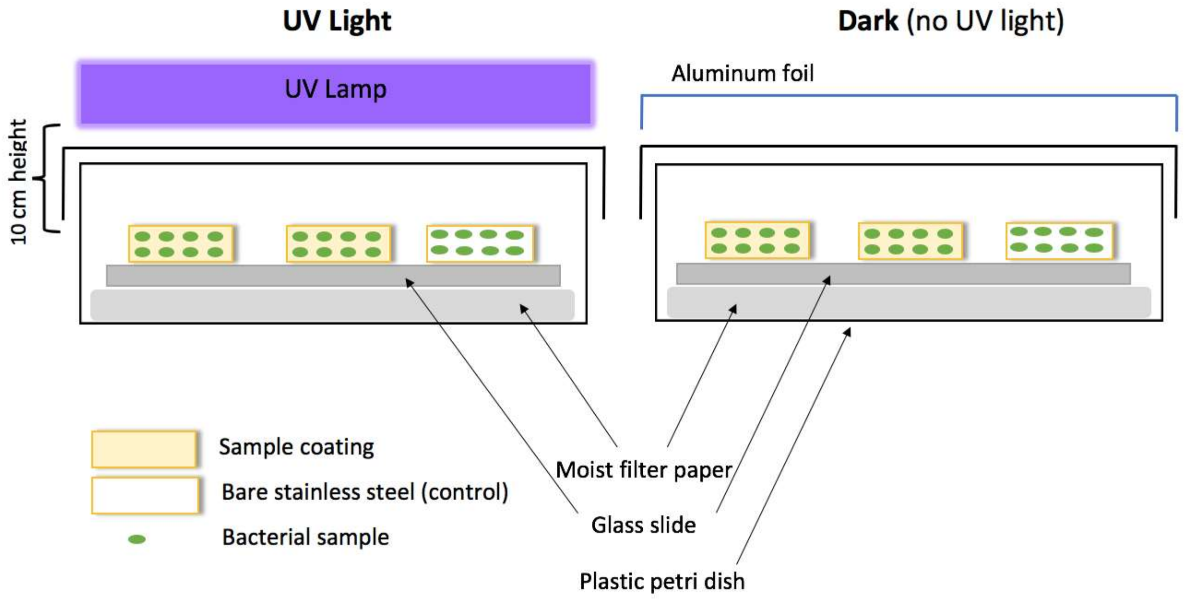


Figure S1. A schematic of the antimicrobial testing environment. Inside each petri dish, a bare stainless steel substrate was kept as a control, while a dark experiment served as a control for each irradiated experiment [Torres Dominguez, E. 2020. Ph.D. Dissertation, University of Missouri].

**Coatings' indents from the screening of factors experiment**

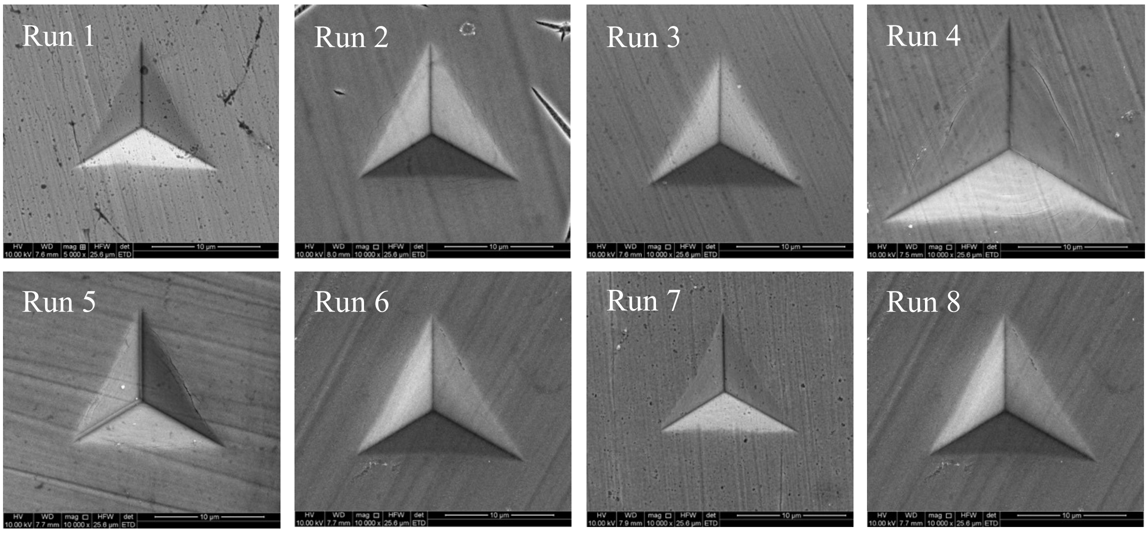


Figure S3. Scanning electron micrographs of typical indents made on samples prepared for the screening of factors experiment. Although the coatings from the eight runs were subjected to the

same final load, their indents' sizes were not the same because each coating had different mechanical properties. Scale bar is 10 µm in all images [Torres Dominguez, E. 2020. Ph.D. Dissertation, University of Missouri].

## Pareto graphs from statistical analysis

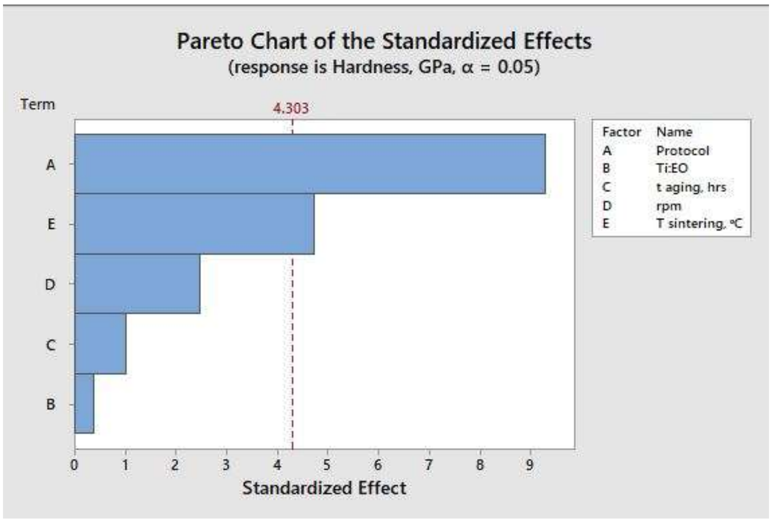


Figure S4. Pareto chart from the screening of factors experiment having coatings' hardness as the experimental response. Observe the relative magnitude of the statistically significant factors protocol type and sintering temperature.

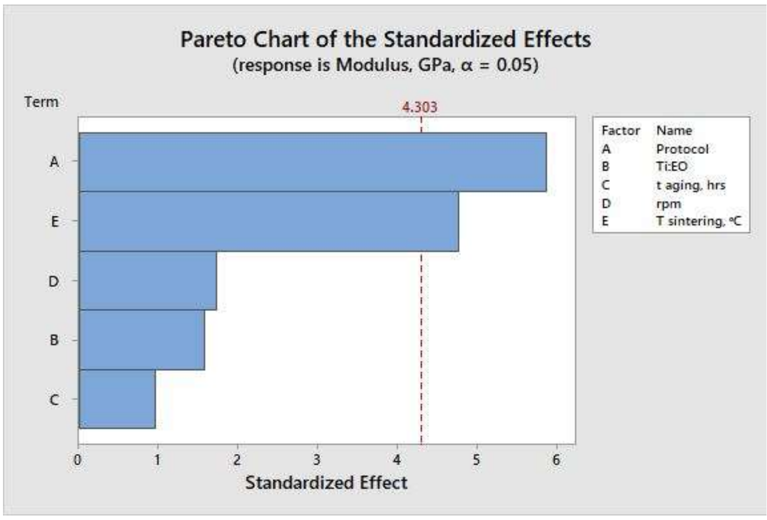


Figure S5. Pareto chart from the screening of factors experiment having coatings' elastic modulus as the experimental response. Observe the relative magnitude of the statistically significant factors protocol type and sintering temperature.
